# Leukocyte Telomere Length Mediates the Associations between Blood Lead and Cadmium with Hypertension among Adults in the United States: A Cross-Sectional Study

**DOI:** 10.3390/toxics12060409

**Published:** 2024-06-03

**Authors:** Changping Ouyang, Yinan Yang, Jinhua Pan, Heming Liu, Xuemei Wang, Shengze Zhou, Xiaoru Shi, Yanxia Zhang, Dan Wang, Xiaobin Hu

**Affiliations:** 1 Institute of Epidemiology and Health Statistics, School of Public Health, Lanzhou University, No. 199 Donggang West Road, Chengguan District, Lanzhou 730000, China; ouyangchp@163.com (C.O.); panjh2017@lzu.edu.cn (J.P.); 220220912581@lzu.edu.cn (H.L.); 220220912900@lzu.edu.cn (X.W.); 220220913251@lzu.edu.cn (S.Z.); shixr2018@lzu.edu.cn (X.S.); 13526745076@163.com (Y.Z.); 18940905659@163.com (D.W.); 2 The Fifth Affiliated Hospital, Sun Yat-sen University, Zhuhai 519000, China; ldyangyn@163.com

**Keywords:** blood lead, blood cadmium, hypertension, leukocyte telomere length (LTL), mediation, NHANES

## Abstract

There is evidence to support the links between lead and cadmium exposure with hypertension and also with leukocyte telomere length (LTL). The objective of this study is to investigate the role that LTL may play in the relationship between lead and cadmium exposure and hypertension. This study consisted of 3718 participants from the National Health and Nutrition Examination Survey (NHANES) 1999–2002. Logistic regression was used to analyze the relationship between blood metals with hypertension, and the mediating model was used to evaluate the mediating effect of LTL. In the fully adjusted model, both blood lead and cadmium ln-transformed concentrations were significantly positively associated with hypertension risk, as were all quartiles of blood lead. Additionally, we observed positive linear dose–response relationships with hypertension by restricted cubic spline analysis (both *p* overall < 0.001, *p* non-linear = 0.3008 for lead and *p* non-linear = 0.7611 for cadmium). The ln-transformed blood lead and cadmium concentrations were associated with shorter LTL. LTL was inversely related to hypertension and the OR was 0.65 (95% CI: 0.47 to 0.89). Furthermore, LTL had mediating effects on the associations of blood lead and cadmium with hypertension risk, and the mediation proportions were 2.25% and 4.20%, respectively. Our findings suggested that exposure to lead and cadmium raised the risk of hypertension, while LTL played as a mediating factor.

## 1. Introduction

Considering a major risk factor for dementia, chronic renal disease, and cardiovascular disorders, hypertension contributes significantly to the worldwide burden of disease [1]. Elevated blood pressure has also been linked to premature death. Between 1990 and 2015, the global death toll related to blood pressure increased dramatically, with an estimated 770 to 10.4 million deaths per year due to elevated blood pressure, and 10.7 million deaths in 2015 attributable to systolic blood pressure ≥ 110–115 mmHg, accounting for 19.2% of all deaths [2]. In 1990, there were 650 million hypertensive individuals aged 30–79; by 2019, that number had risen to 1.28 billion, and the global age-standardized prevalence of hypertension was more than 30%. There have been significant global gaps in the diagnosis, treatment, and disease burden of hypertension over the past three decades [3].

Lead and cadmium are hazardous environmental elements that may have adverse effects on human health. The general public is mostly exposed to tiny amounts of lead, cadmium, and other toxic metals through the following three ways: air inhalation, water and food contaminated toxic metal intake, and skin contact [4]. Exposure to toxic metals leads to oxidative stress, which can result in peroxidation of lipids, modification of proteins, damage to deoxyribonucleic acid (DNA), and other issues [5]. Lead and cadmium exposure have an influence on some diseases, including diabetes [6], hypertension [7], kidney disease [8], and other cardiovascular diseases (CVDs) [9]. As demonstrated by earlier research, exposure to either lead or cadmium elevates blood pressure and raises hypertension risk, and combined exposure is more significantly associated with elevated blood pressure [10,11]. Lanphear B.P. et al. reported that even low-level lead exposure, an important but largely overlooked risk factor, increased the risk of death for CVD [12]. Due to widespread and persistent metal pollution, environmental exposure to toxic metals remains an important public health issue affecting population health.

Telomeres are chromosome structures located at the ends of linear chromosomes that sustain genomic stability by preventing the loss of DNA sequences, avoiding end-to-end chromosome fusion, and distinguishing between DNA breaks and linear ends of chromosomes [13]. Telomerase is a DNA polymerase dependent on ribonucleic acid (RNA), and the biosynthesis of telomeres depends on telomerase [14]. Telomere and telomerase have an important impact on hypertension [15,16]. Previous studies have indicated that leukocyte telomere length (LTL) is an effective indicator of vascular aging and related diseases [17,18]. Exposure to lead and cadmium may induce oxidative stress, which may affect telomere length [19]. Telomere length has been found to be strongly and independently correlated with environmental exposure to cadmium [20]. Thus, exposure to lead and cadmium influences telomere length alterations, which are linked to blood pressure levels. Nevertheless, it is unclear how LTL affects the relationship between blood pressure and exposure to lead and cadmium.

This cross-sectional study was carried out based on data obtained from the National Health and Nutrition Examination Survey (NHANES) for the years 1999–2002. This study aimed to examine the association between lead and cadmium exposure and hypertension and investigate the potential effect of telomere length in the general population of the United States.

## 2. Materials and Methods

### 2.1. Study Population

**Figure 1:**
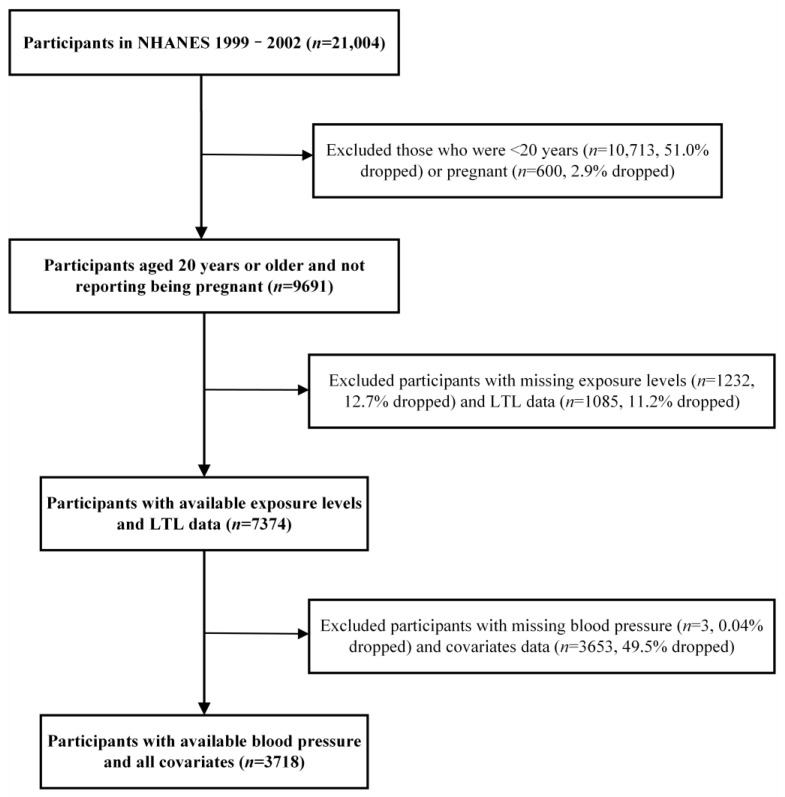
Flow chart of the selection process for eligible participants.

NHANES is a cross-sectional study program to assess the health and nutrition status of adults and children in the United States. Data from the NHANES cycles of 1999–2000 and 2001–2002 were utilized. Among 21,004 subjects who were interviewed, we excluded those aged <20 years (*n* = 10,713) and being pregnant (*n* = 600), without blood lead or cadmium concentrations or LTL (*n* = 2317), and missing covariates (*n* = 3656). This study finally included a total of 3718 participants (Figure 1).

### 2.2. Exposure Measurement 

Blood lead and cadmium concentrations were measured in accordance with published laboratory methods by the National Center for Environmental Health. Quantification of cadmium and lead is based on measuring the amount of light absorbed by ground-state atoms of the metals at 228.8 nm and 283.3 nm, respectively, from sources such as electrodeless discharge lamps (EDLs) or hollow cathode lamps (HCLs). Reconstituted human blood, bovine blood quality control pools, and aqueous standards are diluted using a matrix modifier (ammonium phosphate, triton X-100, and nitric acid). A PerkinElmer Model SIMAA 6000 simultaneous multi-element atomic absorption spectrometer with Zeeman background correction is utilized for assessing the levels of lead and cadmium [21,22]. In the study we conducted, the blood lead was reported in μg/dL and the cadmium in μg/L. The limits of detection (LODs) and distribution of blood lead and cadmium are shown in Supplementary Table S1. Quantities of 0.3 μg/dL and 0.3 μg/L were the LODs for blood lead and blood cadmium, respectively [23]. In total, 0.4% (*n* = 14) and 22.2% (*n* = 826) of the researchers found lower LODs for blood lead and cadmium, respectively. The LOD divided by √2 was used to substitute values that were lower than the LOD.

### 2.3. LTL Measurement

The LTL experiment was performed in Dr. Elizabeth Blackburn’s laboratory. Quantitative polymerase chain reaction (PCR) was applied to quantify LTL relative to standard reference DNA (T/S ratio). To calculate the conversion from T/S ratio to bp, telomeric restriction fragment (TRF) length from Southern blot analysis and T/S ratios were compared using DNA samples from the human diploid fibroblast cell line IMR90 at different population doublings [24,25]. The calculation was 2413 × (T/S) + 3274. 

### 2.4. Outcome Ascertainment

All blood pressures (systolic and diastolic) were measured at the mobile examination center (MEC) by trained staff using a mercury sphygmomanometer on the right arm. After 5 min of calm relaxation, the eligible individuals had blood pressure taken 3 or 4 times [26,27]. The reported average of multiple measurements was used for analysis. If the researcher satisfied at least one of the following requirements, hypertension was taken into consideration: (1) a minimum diastolic blood pressure of 80 mmHg or a minimum systolic blood pressure of 130 mmHg; (2) self-reported physician’s diagnosis of hypertension; (3) currently taking medication for hypertension [28].

### 2.5. Covariates

We selected several covariates by referring to the covariates used in some previous studies on hypertension as potential confounders. All covariates can be divided into sociodemographic status, behaviors related to healthy lifestyle, the presence of other diseases, sex (male or female), age (<65 years or ≥65 years), race/ethnicity (Non-Hispanic White, Non-Hispanic Black, other Hispanic, or other race), marital status (married/living with partner, widowed/divorced/separated, or never married), educational attainment (less than 9th grade, 9–11th grade, high school grade/GED or equivalent, some college or AA degree, or college graduate or above), poverty income ratio (PIR, <1, 1–3, or >3) [29], family history of hypertension (yes or no), smoking status (never, former smoker, or current smoker) [30], alcohol consumption (yes or no), average daily energy intake (≤1527, 1528–2055, 2055–2732, or >2732 kcal), physical activity (none, moderate, or vigorous), body mass index (BMI, ≤25.0, 25.1–29.9, or ≥30.0 kg/m^2^), cardiovascular diseases (CVDs, yes or no), and diabetes (yes or no). According to the U.S. Census Bureau, PIR was an index of socioeconomic status that was calculated by dividing a family’s self-reported income by the poverty guideline the threshold. The definition of a smoker was as follows: a current smoker was one who smokes more than 100 cigarettes in their lifetime; a former smoker was one who smokes more than 100 cigarettes in their lifetime but does not smoke now; and a never smoker never smoked more than 100 cigarettes in their lifetime. The alcohol consumption was categorized by 12 drinks/year [31]. Participating in any kind of moderate or vigorous activity, exercise, or fitness for less than 10 min a week was not considered physical activity. Diabetes was identified by measuring glycosylated hemoglobin [HbA1c] ≥ 6.5%23 or by self-reported current use of insulin or oral hypoglycemic medications [32]. CVD was regarded as the self-reported diagnosis by a physician of heart disease, stroke, angina pectoris, congestive heart failure, and heart attacks [33].

### 2.6. Statistical Analysis

In order to summarize the population’s demographic features, descriptive statistics were used. The continuous variables were expressed as mean ± standard deviation ($\bar{x}$ ± s), the categorical variables were expressed as percentages (%), and we utilized the *t*-test and the Chi-square test to compare the hypertensive and non-hypertensive population.

With three models, binary logistic regression analysis was utilized to explore the association between lead and cadmium exposure and hypertension risk, as well as the association between LTL and hypertension risk. Model 1 did not involve any covariates. Model 2 was adjusted for sex, age, race/ethnicity, marital status, education, poverty/income ratio, family history of hypertension, smoking status, alcohol consumption, average daily energy intake, physical activity, and BMI. Model 3 further incorporated CVD and diabetes. Given the right-skewed distribution of blood lead and cadmium concentrations, metal content was converted to natural logarithm (ln), with a distribution similar to normal. The lowest quartile array was selected as the reference group, and the patients were grouped based on the quartiles of blood lead and blood cadmium concentrations. Then, hypertension OR values and 95% confidence intervals (95% CIs) were calculated. By considering the median of each quartile as a continuous variable in the model, the linear trend test was performed. By fitting three knots (ln-transformed 25th, 50th, and 75th percentiles), limiting the cubic spline in the range among the 5th and 95th percentiles, the dose–response associations between blood lead and blood cadmium levels and the risk of hypertension were further examined in Model 3. Non-linearity was assessed by likelihood ratio tests.

To assess the relationship between lead and cadmium exposure and LTL, linear regression was used. Since LTL was not uniformly distributed, natural ln transformation was applied. The two previously described types of lead and cadmium levels were also utilized to calculate the percent difference and 95% CIs for LTL.

To determine the possible impacts of LTL on the correlations between blood lead and cadmium levels and the risk of hypertension, a model using IE (indirect effect) divided by TE (total effect) was utilized to determine the proportion of mediation. IE was the effect of lead and cadmium exposure on hypertension through LTL, and DE was the effect of lead and cadmium exposure on hypertension without LTL. In the mediation analysis, the quasi-Bayes Monte Carlo method was used, and 1000 simulations were performed based on the normal approximation.

All statistical analyses were performed with R software (version 4.1.3, Copyright The R Foundation for Statistical Computing). A two-sided *p* value of <0.05 was considered statistically significant.

## 3. Results

### 3.1. Characteristics of Participants

Supplementary Table S2 shows the demographic characteristics of participants with and without hypertension. The population was 56.8% male and 43.2% female. There were 1907 participants with hypertension (51.3%) among the 3718 participants aged over 20 years, and 60.2% were men. With the exception of PIR, there were statistical differences between participants with and without hypertension in terms of sex, age, race/ethnicity, marital status, educational attainment, smoking status, alcohol consumption, average daily energy intake, physical activity, BMI, CVD, and diabetes. In comparison to non-hypertension patients, hypertensive participants exhibited shorter LTL and higher concentrations of lead and cadmium.

### 3.2. Blood Metal Concentrations and Hypertension Risk

To examine the relationships between lead and cadmium levels and the risk of hypertension, we employed three different models. The crude and adjusted ORs (95% CIs) of hypertension for blood lead and cadmium concentrations are summarized in Table 1. We observed that there was a statistically significant correlation between ln-transformed blood lead concentrations and blood pressure in every model. The risk of hypertension was higher in the other quartiles of blood lead levels than in the first quartile (both *p* < 0.05). As a comparison to the reference group, the calculated ORs (95% CIs) of hypertension in Model 3 were 1.40 (1.14 to 1.72), 1.52 (1.23 to 1.89), and 2.02 (1.59 to 2.56) for the second to fourth quartiles of blood lead levels (*p* value for trend < 0.001). As for ln-transformed blood cadmium concentration, no significant association was found in Model 1, and significantly positive associations were found after adjusting by covariates (Model 2 and Model 3). In Model 3, the calculated ORs (95% CIs) of hypertension correlates with the blood cadmium levels’ second to fourth quartiles were 1.10 (0.89 to 1.36), 1.62 (1.32 to 2.00), and 1.54 (1.18 to 2.01) when compared to the reference group (*p* value for trend < 0.001).

**Figure 2:**
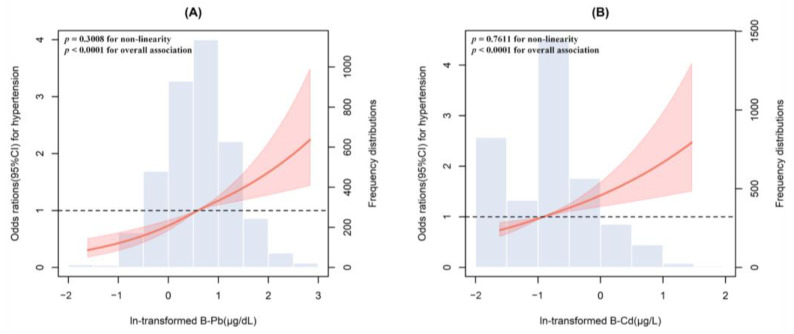
Adjusted restricted cubic spline for the association between blood lead (**A**) and cadmium (**B**) with hypertension. Model adjusted for sex, age, race/ethnicity, marital status, education, poverty/income ratio, family history of hypertension, smoking status, alcohol consumption, average daily energy intake, physical activity, BMI, CVD, and diabetes.

The multivariate-adjusted restricted cubic spline analyses showed positive associations with the hypertension risk for both ln-transformed blood lead and cadmium concentrations (all *p* for overall < 0.0001, *p* for non-linearity of 0.3008 and 0.7611, respectively) (Figure 2).

### 3.3. Blood Metal Concentrations and LTL

**Figure 3:**
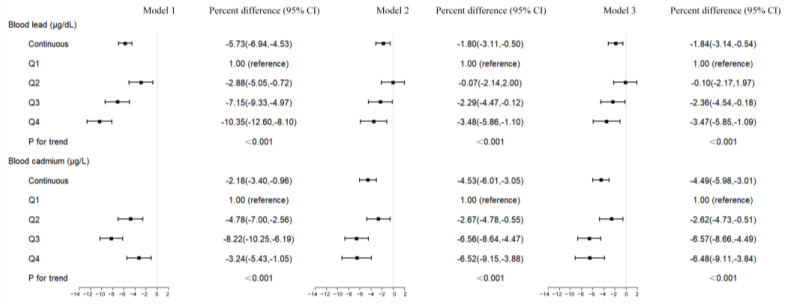
Percent difference (95% CIs) in LTL by lead and cadmium exposure.

Based on linear regression, Figure 3 displays the percent difference (95% CIs) of LTL for blood lead and cadmium concentrations. We discovered statistically significant negative relationships for both ln-transformed blood lead and cadmium contents in all models. In three models, the LTL of the second to fourth quartiles of blood cadmium levels was lower than that of the reference group (all *p* < 0.05). As for blood lead levels, we found the same result only in the unadjusted model (Model 1) (both *p* < 0.05). The second quartile showed no significant correlation with LTL once variables were taken into account (Model 2 and Model 3), while the third and fourth quartiles did.

### 3.4. LTL and Hypertension Risk

Table 2 displays the associations of LTL and hypertension risk by logistic regression. We observed that hypertension risk was decreased with increasing LTL in all models (both *p* < 0.05). The estimated ORs (95% CIs) of hypertension associated with LTL in three models were 0.26 (0.19 to 0.34), 0.62 (0.45 to 0.85), and 0.65 (0.47 to 0.89), respectively.

### 3.5. Mediation Analyses

**Figure 4:**
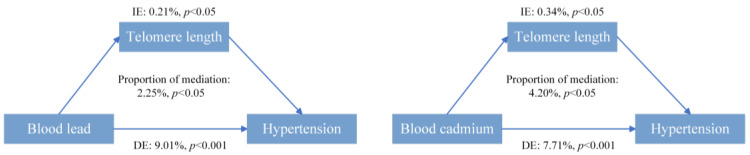
Estimated proportion of the association between metals and hypertension.

Furthermore, for the purpose of evaluating the potential effects of LTL, we performed a simple mediation effect analysis. The findings demonstrated that correlations between exposure to lead and cadmium with the development of hypertension were significantly mediated by LTL. The value of mediating effects was 0.0021 and 0.0034, and the proportion of mediation was 2.25% and 4.20%, respectively (Figure 4).

## 4. Discussion

In our study, we discovered that blood lead and cadmium had substantially positive relationships with hypertension in the fully adjusted model. For every 1 μg/dL higher blood lead concentration and every 1 μg/L higher blood cadmium concentration, the risk of hypertension increased by 46% and 35%, respectively. Those with blood lead and cadmium concentrations in the highest quartiles had about 102% and 54% higher hypertension risk than those in the lowest quartile. Moreover, exposure to lead and cadmium and hypertension risk may be mediated by LTL.

The findings of this study are in line with earlier research on the connection between exposure to lead and cadmium and hypertension. Wu et al. declared that blood lead and cadmium had positive relationships with hypertension risk and also with diastolic and systolic pressure [34]. According to a study utilizing the NHANES 1999–2018 database, hypertension prevalence rose by 12% in the group with the highest blood lead concentration quartiles compared that with the lowest quartiles [35]. Meanwhile, the same association was shown between blood cadmium and hypertension risk in a study based on the KNHANES (Korean National Health and Nutrition Examination Survey) database [11]. Furthermore, it was reported that blood cadmium had a stronger association than lead with elevated blood pressure and hypertension risk in research based on the KNANES [10].

Lead and cadmium are hazardous substances that exist widely in the environment and have various adverse effects on human health [5]. Although the mechanisms of lead and cadmium toxicity for humans are not yet clear, several studies have explored some possible mechanisms. Lead and cadmium may induce endothelial dysfunction, specifically by enhancing the generation of reactive oxygen species, which can decrease nitric oxide bioavailability, increase vasoconstrictive activity, and eventually lead to the occurrence and development of hypertension [19,36,37]. Lead and cadmium also cause dysregulation of the renin–angiotensin–aldosterone system and increase levels of norepinephrine, which affects vascular smooth muscle, thus promoting vascular constriction [36,38]. Furthermore, there are idiosyncratic mechanisms in lead-induced hypertension. Lead is a calcium-like element that competes with calcium for transportation through endoplasmic reticulum channels and pumps, which impacts vascular resistance [19,37].

Consistent with earlier studies, a significantly negative association was found between blood cadmium and LTL. A prior study based on the NHANES reported that a doubled increase in blood cadmium was linked to a 2.46% reduction in LTL and the highest quartile of blood cadmium was associated with 5.54% shorter LTL compared with the lowest quartile [20]. In contrast, no association was found between blood lead and LTL [20]. In agreement with the above research, Wu et al. reported the same results about blood lead and LTL [39]. However, it is worth noting that a significantly negative association was also found between blood lead and LTL in our study, which was different from previous studies. A prospective U.S. prebirth cohort observed that exposure to lead and cadmium during pregnancy were inversely related to newborn relative LTL, whereas our participants were non-pregnant [40]. The effects of lead and cadmium on LTL might be induced through inflammation and oxidative stress, which are caused by an imbalance in the generation of reactive oxygen species (ROS) and detoxification [41,42]. All in all, further research is needed on the relationship between lead exposure and LTL.

A negative association was observed between LTL and hypertension risk, in line with some previous studies. Cheng et al., for example, reported that the LTL of essential hypertension patients was significantly shorter, which might be due to the defective TERT (telomerase reverse transcriptase) and TERC (internal RNA template) expression in leukocytes, which is responsible for maintaining and prolonging LTL and plays a role in regulating tissue repair and regeneration [43]. This was also reported in a community-based study in Lebanon and a study about coal miners in China [44,45]. The mechanism by which telomere length causes hypertension is still unclear. In addition to the above mentioned possible mechanism inferred by Chen et al., other possible mechanisms are summarized in a review [46]. Firstly, LTL shortening may lead to cellular senescence. LTL shortening accelerates the aging process of vascular endothelial cells, increases cholesterol deposition, forms plaque, and then leads to atherosclerosis [47]. Aging vascular smooth muscle cells (VSMCs) in atherosclerotic plaques are limited in their ability to proliferate and have increased matrix-degrading enzyme activity that promotes fiber cap thinning and plaque rupture, which can lead to subsequent thrombosis, myocardial infarction, or stroke [46]. In addition, the repair mechanism of vascular atherosclerosis relies on endothelial progenitor cells (EPCs), and LTL shortening limits the number and function of EPCs, which in turn impair the replication potential of the damaged part of the vasculature [46,48]. Secondly, the shortening of LTL reduces the regenerative ability of cardiomyocytes, and the accumulation of old dead cells eventually leads to cardiac pumping failure [49]. Finally, shortening of LTL decreases leukocyte function and causes inflammatory response. During this process, a large number of CD8^+^ T cells are present, and their secretion of tumor necrosis factor-α (TNF-α) and interleukin-6 (IL-6) causes low-grade systemic inflammation, which is a pathogenic factor for atherosclerosis and cardiovascular diseases [42]. However, serval studies have reported different conclusions. A Mendelian randomization study declared that LTL had a positive association with hypertension risk, which was opposite to the other CVDs, such as myocardial infarction, ischemic heart disease, coronary atherosclerosis, and stroke [50]. Additionally, it has also been reported that there was no association between LTL and blood pressure or hypertension risk [51,52]. The relationship between LTL and hypertension was uncertain, and the current conclusions about it were completely contradictory. Hence, it is necessary to conduct more research to explore its specific relationship.

In mediation analysis, LTL had mediating effects on the associations of blood lead and cadmium with hypertension risk, even though the mediation proportions were only 2.25% and 4.20%, respectively. This finding suggested that in addition to their direct effects on the development and progression of hypertension, blood lead and cadmium may also have indirect effects by accelerating cell senescence. This mediating effect of LTL has been demonstrated in other diseases or indicators to assess health status. Chen et al. observed that LTL mediated the relationships between urinary metals and osteoarthritis risk, with a mediated proportion of 9.81% [53]. Zhang et al. found the same in the relationships between lead and cadmium with blood glucose [54], and Li et al. also found it in the relationship between lead and eGFR (glomerular filtration rate) [55]. To our knowledge, this may be the first study based on the NHANES database to examine the mediating role of LTL on the associations between blood metals and hypertension risk. 

This study has several strengths, as follows: Firstly, a substantial inverse correlation between blood lead and LTL was discovered, which was different from previous studies. Additionally, the subjects in this study were from a non-occupationally exposed population that was exposed to low doses, making it possible to investigate the consequences of low levels of exposure.

There are also some limitations to this study. First, its cross-sectional nature makes it impossible to infer causality. Second, the concentration of blood lead and cadmium measured at one time may not fully reflect the actual exposure, and it is affected by metabolism. Third, even though we adjusted for confounding factors from three aspects, we were not able to completely adjust for all confounding factors, such as unknown or residual confounding factors.

## 5. Conclusions

In summary, our study revealed a link between lead and cadmium exposure with a higher risk of hypertension as well as a lower LTL. Moreover, LTL shortening was related to increased hypertension risk. On the basis of the above findings, mediation analyses suggested that LTL may be a mediating factor in the association between exposure to lead and cadmium and hypertension. The exact mechanism behind these associations is still unclear, and it is critical that more investigation into the mechanisms is carried out in the future in order to better explain these associations.

## Figures and Tables

**Table 1:** Odds ratios (95% CIs) for hypertension associated with blood lead and blood cadmium concentrations, NHANES 1999–2002.

Blood Metals	Continuous	Q1	Q2	Q3	Q4	*p* for Trend *
Blood lead (μg/dL)		≤1.2	1.2–1.8	1.8–2.8	>2.8	
Model 1	1.71 (1.54, 1.90)	1.00 (reference)	1.56 (1.30, 1.88)	1.93 (1.60, 2.33)	2.66 (2.19, 3.24)	<0.001
Model 2	1.43 (1.26, 1.63)	1.00 (reference)	1.38 (1.13, 1.70)	1.48 (1.20, 1.83)	1.96 (1.55, 2.48)	<0.001
Model 3	1.46 (1.28, 1.66)	1.00 (reference)	1.40 (1.14, 1.72)	1.52 (1.23, 1.89)	2.02 (1.59, 2.56)	<0.001
Blood cadmium (μg/L)		≤0.3	0.3–0.4	0.4–0.7	>0.7	
Model 1	1.10 (0.99, 1.22)	1.00 (reference)	1.19 (0.98, 1.44)	1.65 (1.39, 1.97)	1.13 (0.94, 1.36)	0.004
Model 2	1.34 (1.16, 1.55)	1.00 (reference)	1.11 (0.90, 1.37)	1.60 (1.30, 1.97)	1.53 (1.17, 1.99)	<0.001
Model 3	1.35 (1.16, 1.57)	1.00 (reference)	1.10 (0.89, 1.36)	1.62 (1.32, 2.00)	1.54 (1.18, 2.01)	<0.001

* *p* for trend test was performed with the median value of each quartile treated as a continuous variable in the models. Model 1: unadjusted for any covariates. Model 2: adjusted for sex, age, race/ethnicity, marital status, education, poverty/income ratio, family history of hypertension, smoking status, alcohol consumption, average daily energy intake, physical activity, and BMI. Model 3: Model 2 plus self-reported CVD and diabetes.

**Table 2:** Odds ratios (95% CIs) for hypertension associated with LTL, NHANES 1999–2002.

Leukocyte Telomere Length	OR	95% CI	*p* Value
Model 1	0.26	0.19, 0.34	<0.001
Model 2	0.62	0.45, 0.85	0.003
Model 3	0.65	0.47, 0.89	0.007

## Data Availability

The data are publicly available on the NHANES website: https://www.cdc.gov/nchs/nhanes/Index.htm (accessed on 24 April 2024).

## Supplementary Materials

The following supporting information can be downloaded at: https://www.mdpi.com/article/10.3390/toxics12060409/s1, Table S1: Limits of detection (LODs) and distributions for blood lead and cadmium among participants in NHANES 1999–2002 (*n* = 3718); Table S2: Basic characteristics of participants among U.S. adults, NHANES 1999–2002.
