# Supplementary material for: Leukocyte Telomere Length Mediates the Associations between Blood Lead and Cadmium with Hypertension among Adults in the United States: A Cross-Sectional Study
Source: Toxics. 2024 Jun 3;12(6):409. doi: 10.3390/toxics12060409 (PMC11209134; doi:10.3390/toxics12060409)
Supplement: Supplementary file 1 [file toxics-12-00409-s001.zip › toxics-3003724-supplementary.pdf]

Supplementary Table S1 Limits of detection (LOD) and distributions for blood lead and cadmium among participants in NHANES 1999-2002 (n = 3718)

Metals	LOD	N (%) < LOD	Minimum	25th percentile	50th percentile	75th percentile	Maximum
Blood lead	0.3 μg/dL	14 (0.4%)	0.2	1.2	1.8	2.8	54.0
Blood cadmium	0.3 μg /L	826 (22.2%)	0.2	0.3	0.4	0.7	8.5

* The units of distributions for blood metal: blood lead (μg/dL); blood cadmium (μg/L).

For values below the limit of detection were calculated using the detection limit for metals divided by the square root of two.

Supplementary Table S2 Basic characteristics of participants among U.S. adults, NHANES 1999–2002

Characteristics	Total (n=3718)	Non-hypertension (n=1811)	With hypertension (n=1907)	*P* Value
Sex				
male	2112 (56.8)	964 (53.2)	1148 (60.2)	<0.001
female	1606 (43.2)	847 (46.8)	759 (39.8)	
Age, years old				
< 65	2971 (79.9)	1651 (91.2)	1320 (69.2)	<0.001
≥ 65	747 (20.1)	160 (8.8)	587 (30.8)	
Race/ethnicity				
Mexican American	852 (22.9)	481 (26.6)	371 (19.5)	<0.001
Non-Hispanic White	2045 (55.0)	974 (53.8)	1071 (56.2)	
Non-Hispanic Black	547 (14.7)	212 (11.7)	335 (17.6)	
Other race	274 (7.4)	144 (8.0)	130 (6.8)	
Marital status				
Married/Living with partner	2430 (65.4)	1170 (64.6)	1260 (66.1)	<0.001
Widowed/Divorced/Separated	695 (18.7)	255 (14.1)	440 (23.1)	
Never married	593 (15.9)	386 (21.3)	207 (10.9)	
Education				
Less Than 9th Grade	435 (11.7)	188 (10.4)	247 (13.0)	<0.001
9-11th Grade	581 (15.6)	261 (14.4)	320 (16.8)	
High School Grad/GED or Equivalent	881 (23.7)	406 (22.4)	475 (24.9)	
Some College or AA degree	1000 (26.9)	520 (28.7)	480 (25.2)	
College Graduate or above	821 (22.1)	436 (24.1)	385 (20.2)	
Poverty income ratio				

< 1	526 (14.1)	274 (15.1)	252 (13.2)	0.093
1-3	1430 (38.5)	669 (36.9)	761 (39.9)	
> 3	1762 (47.4)	868 (47.9)	894 (46.9)	
Family history of hypertension				
No	2608 (70.1)	1309 (72.3)	1299 (68.1)	0.006
Yes	1110 (29.9)	502 (27.7)	608 (31.9)	
Smoking status				
Never smoker	1683 (45.3)	872 (48.2)	811 (42.5)	<0.001
Former smoker	1025 (27.6)	402 (22.2)	623 (32.7)	
Current smoker	1010 (27.2)	537 (29.7)	473 (24.8)	
Alcohol consumption				
No	1891 (50.9)	839 (46.3)	1052 (55.2)	<0.001
Yes	1827 (49.1)	972 (53.7)	855 (44.8)	
Average daily energy intake				
Q1 (≤ 1527kcal)	930 (25.0)	398 (22.0)	532 (27.9)	<0.001
Q2 (1528-2055kcal)	929 (25.0)	433 (23.9)	496 (26.0)	
Q3 (2055-2732kcal)	929 (25.0)	457 (25.2)	472 (24.8)	
Q4 (> 2732kcal)	930 (25.0)	523 (28.9)	407 (21.3)	
Physical activity				
None	1389 (37.4)	617 (34.1)	772 (40.5)	<0.001
Moderate	1795 (48.3)	895 (49.4)	900 (47.2)	
Vigorous	534 (14.4)	299 (16.5)	235 (12.3)	
BMI, kg/m^2				
≤ 25.0	1298 (34.9)	790 (43.6)	508 (26.6)	<0.001
25.1-29.9	1359 (36.6)	640 (35.3)	719 (37.7)	
≥ 30.0	1061 (28.5)	381 (21.0)	680 (35.7)	
With CVD				

No	3409 (91.7)	1734 (95.7)	1675 (87.8)	<0.001
Yes	309 (8.3)	77 (4.3)	232 (12.2)	
With diabetes				
No	3377 (90.8)	1731 (95.6)	1646 (86.3)	<0.001
Yes	341 (9.2)	80 (4.4)	261 (13.7)	
LTL	1.00 [0.85, 1.17]	1.04 [0.89, 1.21]	0.96 [0.81, 1.13]	<0.001
Blood lead, μg/dL	1.80 [1.20, 2.80]	1.60 [1.10, 2.40]	2.10 [1.40, 3.10]	<0.001
Blood cadmium, μg/L	0.40 [0.30, 0.70]	0.40 [0.20, 0.60]	0.50 [0.30, 0.70]	<0.001
